# The Impact of Collaborative Documentation on Person-Centered Care: Textual Analysis of Clinical Notes

**DOI:** 10.2196/52678

**Published:** 2024-09-20

**Authors:** Victoria Stanhope, Nari Yoo, Elizabeth Matthews, Daniel Baslock, Yuanyuan Hu

**Affiliations:** 1 Silver School of Social Work, New York University, 1 Washington Square N, New York, NY, 10003, United States, 1 3016931203; 2 Graduate School of Service, Fordham University, New York, NY, United States; 3 School of Social Work, Virginia Commonwealth University, Richmond, VA, United States; 4 School of Social Work, University of Minnesota, St Paul, MN, United States

**Keywords:** person-centered care, collaborative documentation, natural language processing, concurrent documentation, clinical documentations, visit notes, community, health center, mental health center, textual analysis, clinical informatics, behavioral health, mental health, linguistic, linguistic inquiry, dictionary-based, sentence fragment, psychology, psychological, clinical information, decision-making, mental health services, clinical notes, NLP

## Abstract

**Background:**

Collaborative documentation (CD) is a behavioral health practice involving shared writing of clinic visit notes by providers and consumers. Despite widespread dissemination of CD, research on its effectiveness or impact on person-centered care (PCC) has been limited. Principles of PCC planning, a recovery-based approach to service planning that operationalizes PCC, can inform the measurement of person-centeredness within clinical documentation.

**Objective:**

This study aims to use the clinical informatics approach of natural language processing (NLP) to examine the impact of CD on person-centeredness in clinic visit notes. Using a dictionary-based approach, this study conducts a textual analysis of clinic notes from a community mental health center before and after staff were trained in CD.

**Methods:**

This study used visit notes (n=1981) from 10 providers in a community mental health center 6 months before and after training in CD. LIWC-22 was used to assess all notes using the Linguistic Inquiry and Word Count (LIWC) dictionary, which categorizes over 5000 linguistic and psychological words. Twelve LIWC categories were selected and mapped onto PCC planning principles through the consensus of 3 domain experts. The LIWC-22 contextualizer was used to extract sentence fragments from notes corresponding to LIWC categories. Then, fixed-effects modeling was used to identify differences in notes before and after CD training while accounting for nesting within the provider.

**Results:**

Sentence fragments identified by the contextualizing process illustrated how visit notes demonstrated PCC. The fixed effects analysis found a significant positive shift toward person-centeredness; this was observed in 6 of the selected LIWC categories post CD. Specifically, there was a notable increase in words associated with achievement (β=.774, *P*<.001), power (β=.831, *P*<.001), money (β=.204, *P*<.001), physical health (β=.427, *P*=.03), while leisure words decreased (β=−.166, *P*=.002).

**Conclusions:**

By using a dictionary-based approach, the study identified how CD might influence the integration of PCC principles within clinical notes. Although the results were mixed, the findings highlight the potential effectiveness of CD in enhancing person-centeredness in clinic notes. By leveraging NLP techniques, this research illuminated the value of narrative clinical notes in assessing the quality of care in behavioral health contexts. These findings underscore the promise of NLP for quality assurance in health care settings and emphasize the need for refining algorithms to more accurately measure PCC.

## Introduction

Collaborative documentation (CD) is a specified behavioral health practice where clinicians complete visit notes jointly with consumers during the session [1]. Through deliberate clinical strategies, such as sharing the computer screen, reading visit notes aloud, and actively seeking consumer’s input into the content of the session note [2], CD is a person-centered strategy that aims to engage and empower individuals and facilitate a mutual agreement on treatment progress, service goals, and session activities. Both as a means to promote person-centered care (PCC) and make health information more accessible and transparent to consumers, the practice of CD is being widely disseminated through formal and informal training for community mental health providers [3].

PCC, also referred to as patient-centered care, is a paradigm shift in health care that is defined by the Institute of Medicine as care that is responsive to individual client preferences, needs, and values [4]. A key part of behavioral health reform, PCC moves away from disease-centered treatment to a more holistic approach that engages individuals as active, empowered partners in their care. Person-centered care planning (PCCP) is a recovery-oriented practice that integrates principles of PCC into the service planning process. PCCP orients service planning and documentation to the unique personal life goals of the consumer [5], and provides a framework for operationalizing PCC in practice through six core principles, which are as follows: (1) PCC is based on the person’s own unique life goals and aspirations; (2) PCC is oriented toward promoting recovery rather than only minimizing illness and symptoms; (3) PCC articulates the person‘s own role and the role of both paid practitioners and natural supports in assisting the person to achieve his or her own goals; (4) PCC focuses and builds on the person’s capacities, strengths, and interests; (5) PCC emphasizes the use of natural community settings rather than segregated program settings; and (6) PCC anticipates and allow for uncertainty, setbacks, and disagreements as inevitable steps on the path to greater self-determination [6].

While CD has emerged as a recognized person-centered practice strategy by fully engaging the consumer in decisions about their care, there remains a very limited evidence base demonstrating its clinical effectiveness, including its impact on PCC. Existing research demonstrates that CD is aligned with consumer preferences; a recent scoping review found that the ability to read their visit notes improved consumers’ experience in mental health care, including their ability to remember their plans of care, understand their treatment, and trust decisions made with providers [7]. In community mental health, the use of CD specifically has been found to strengthen the therapeutic alliance [8], and improve service engagement, both in terms of visit attendance and medication adherence [1].

Despite this preliminary support for CD, more work is needed to examine its impact on quality of care. Adding urgency to this knowledge gap, regulatory changes to the 21st Century Cures Act now mandate organizations to make electronic health and mental health information, including many types of visit notes, accessible online to service users [9]. As a consequence, best practices for using visit notes to support the provision of high-quality PCC are needed.

Clinical informatics, which provides highly efficient ways to mine data within the electronic health record (EHR), is a promising methodological approach to examining the impact of CD on clinical quality. Although behavioral health has lagged behind medical settings in the adoption of EHRs, now the majority of behavioral health settings document visit notes via the EHR [10]. The shift from paper records to EHRs provides an unprecedented opportunity for clinical informatics to inform quality improvement in behavioral health care. Visit notes include nuanced information about care processes, session content, provider perspective, and the consumer experience that are not captured in other more structured fields of the EHR, offering valuable insight into clinical quality that has not yet been systematically targeted in mental health services research [11]. Researchers have applied manual content analysis to visit notes to evaluate dimensions of PCC [12,13], but these efforts are inevitably limited in scope and dependent on the interpretive lens of the researcher [14].

One clinical informatics strategy that can parse large volumes of unstructured narrative information into quantitative data is natural language processing (NLP) [15]. While some text mining approaches use words as the unit of analysis, NLP is able to capture the complexity of unstructured narrative using underlying metadata, which examines how words relate to each other in a sentence and the semantic context of a sentence [16]. The method involves syntactic processing, information extraction, and capturing meaning and relationships across concepts. NLP has been predominantly used for detecting pathology and predicting behavior [17] including measuring the following: alcohol misuse in trauma patients [18], suicidal behavior [19], adverse childhood experiences among VA patients [20], smoking status [21], and sentiment at discharge [22]. Recent studies have used this method to measure quality and safety in nursing care [23], identify integrated care elements within primary care [24], and detect changes in clinical documentation after opening notes to service users [25], however, there have been fewer studies that have used NLP to analyze indicators of mental health care quality, including PCC. By providing a framework to systematically categorize and compare the contents of clinical notes, NLP is well poised to provide novel insight into how CD affects clinical quality and PCC.

The dictionary-based approach in NLP is a method that uses a predefined lexicon to identify and extract certain types of words or phrases within a given text. This approach is often used in tasks such as part-of-speech tagging, named entity recognition, and sentiment analysis. In the dictionary-based approach, the dictionary consists of a list of words or phrases along with their associated tags or labels. For example, in part-of-speech tagging, the dictionary might contain a list of common nouns, verbs, and adjectives, each with a corresponding tag that indicates the word’s part of speech. One of the benefits of the dictionary-based approach is its simplicity and ease of implementation, as it targets only a predefined lexicon, or set of words, and does not require the use of complex algorithms or machine learning models. One of the most dictionary-based approaches is LIWC (Linguistic Inquiry and Word Count), and LIWC analysis has been found to be particularly useful for identifying and analyzing the emotional state of individuals in mental health–related text data [26]. Despite its potential, such as its application in oncology settings to examine the changes in clinical notes after patient access [27], the dictionary-based approach has not yet been applied to clinical notes in behavioral health care settings for its change after CD.

This study examines the effect of CD on the person-centeredness of documentation within a community mental health setting using a quasi-experimental pretest-posttest design. The study adapted a well-established dictionary to conduct sentiment analysis of provider clinic visit notes before and after providers were trained in CD.

## Methods

### Ethical Considerations

This study (IRB-FY2022-5838) was granted an exemption by the New York University Institutional Review Board.

### Data Source

The study setting was a community mental health center, which provided a range of services to people with severe mental illnesses including outpatient therapy, assertive community treatment, community support programs, and psychiatric rehabilitation. The data set, or corpus, is visit notes completed by providers trained in CD. Providers were trained in CD by MTM Services [28], a leader in CD training. Their training consisted of tailored in-person workshops, technical assistance, and a “train the trainer” series designed for practice sustainability. The clinic training consisted of 8 hours of web-based training and customized consultation support for implementation.

The visit notes were clinical narrative documents completed when the provider had an in-person contact with a service user. Visit notes document the following: (1) sessions focused on developing or revising the service plan, a narrative clinical document completed every 6 months with the service user detailing goals, strengths and barriers, short-term objectives, supports, professional/ billable services, and natural support and self-directed actions, and (2) a visit focused on progress made towards completing the steps in the service plan. Inclusion criteria for providers were that they were: full-time employees of the clinic; provided services to adults with severe mental illnesses; trained in CD; and had been employed in their position a year prior and the year after being trained.

This study sampled all visit notes completed by participating providers 6 months prior to CD training and all visit notes completed during the sixth month following training (with a month lag for implementation time) between July 1, 2015, and March 10, 2020. Based on anticipated documentation rates of a clinic note being generated by each visit, we recruited 10 providers, generating a total of 1981 visit notes. On average, 198.1 notes were included per provider with a standard deviation of 37.8. Sampled notes were deidentified but linked to providers through unique identifiers. In addition to the session narrative, each note was comprised of the following sections: (1) Provider ID, (2) Time and Date of Service, (3) Therapy Modality, and (4) Length of Stay.

### Analytic Strategy

We used LIWC-22 to compute the scores for PCC-related sentiment and linguistic categories [29] in each sampled note. The LIWC method is a text analysis tool that uses linguistic algorithms to identify and categorize words in a text according to their psychological properties. The method is based on the LIWC dictionary, which contains over 5000 words and word stems organized into linguistic and psychological categories. In this study, 3 domain experts used a consensus approach to select LIWC categories that mapped onto the 6 principles of PCC [6] with several of the categories mapping onto more than one domain. This iterative process involved 8 rounds of independent coding by 3 raters using Excel, followed by discussions to resolve discrepancies and achieve consensus on the final coding of all data points. According to the Cohen κ measure, raters had an average interrater agreement of 88% across the LIWC categories [30]. Out of a possible 107 LIWC categories, we selected 12 categories. The selected LIWC domains and their associated PCC principles are summarized in Tables 1 and 2 [6,^30^].

**Table 1.** Mapping PCCP^a^ principles onto LIWC^b^ categories.

PCCP principles	LIWC categories
PCCP is based on the person’s own unique life goals and aspirations.	Lifestyle (leisure, home, work, money, religion), social referents (family, friend)
PCCP is oriented toward promoting recovery rather than only minimizing illness and symptoms.	Health (physical, wellness)
PCCP articulates the person’s own role and the role of both paid practitioners and natural supports in assisting the person to achieve his or her own goals.	Social referents (family, friend), physical (physical, wellness)
PCCP focuses and builds on the person’s capacities, strengths, and interests.	Drives (achievement, affiliation, power), lifestyle (leisure, home, work, money, religion)
PCCP emphasizes the use of natural community settings rather than segregated program settings.	Lifestyle (leisure, home, work, money, religion)
PCCP anticipates and allows for uncertainty, setbacks, and disagreements as inevitable steps on the path to greater self-determination.	Drives (affiliation, achievement, power)

a PCCP: person-centered care planning.

b LIWC: Linguistic Inquiry and Word Count.

**Table 2.** Sample fragments from clinic notes generated by contextualizing LIWC^a^ subcategories.

LIWC subcategories		Clinic note excerpt
**Drives**		
	Achievement	Was able to identify her strengths, abilities, and self-identified progress in therapy reports that she has been engaging more socially with friends and that she has been trying to express herself
	Affiliation	Unexpected death of her pet was huge stressor that triggers increases in intensity of depression improvement with community resources and social networking due to becoming more integrated with his new community and within his daughter’s school district
	Power	The client reports with a positive outlook that she feels more in control and is excited to receive praise for using her therapy learning Had no outbursts or over reactions recently and feels proud of her assertive but in control manner
**Lifestyle**		
	Home	Highlighting that he doesn’t like the apartment “being so quiet” when his son is gone… The client has increased productivity at home with baking and wrapping presents.
	Leisure	She is also making self-care more of a priority, “I scheduled a cruise and it’s just my sister and I going” Progress is that he has begun basketball
	Money	She admits that she has no savings of her own but she knows that she will get alimony He has figured out a plan to pay for housing
	Religion	Topics of no control include people’s religious actions and beliefs, elements within his own church and community, as well as the political culture She reported that she has been supported by her church and increased her faith significantly
	Work	He continues to apply for jobs and is now working with workforce development. Not working currently, sent about a couple of job applications, continues with college course work
**Physical**		
	Physical	Strengthen his tongue and swallowing skills, will occur to help reduce his concern regarding health issues Barriers to maintaining treatment plans goals, because her varying blood sugars have caused severe mood swings
	Wellness	Emily is making progress in her goals to increase positive self-worth and applying healthy coping skills Trouble with motivation at times and needing to clear his mind, discussed option of yoga and mindfulness
**Social referents**		
	Family	Has been spending time with her family and working to express her feelings and needs when appropriate Progress is that she has figured out how to resolve some of her parenting issues
	Friend	States that she feels she can “cut loose and have fun” with her friends Regards to her recent trip to [place] which she really enjoyed she made new friends

a LIWC: Linguistic Inquiry and Word Count.

### Analysis

Using the LIWC categories described above, we compared notes pre and post CD training to examine differences in PCC. The complete visit note was used as the unit of analysis. For data preprocessing, we cleaned the data and converted the information into a structured format that made it amenable to identifying patterns in the data. The LIWC-22 dictionary is case-insensitive and allows for matching 2-word phrases. The LIWC-22 software removes extra whitespace characters by default. While irrelevant words are not explicitly defined in the dictionary, we removed section headings (eg, Location) from the clinical notes before processing the text to eliminate some irrelevant words. Negated phrases (eg, “not happy”) are not treated differently from nonnegated phrases in the standard scoring. To address this limitation, we included an additional analysis in Multimedia Appendix 1 that controls for the negations score.

To validate the team’s selection of LIWC categories, we first used the Contextualizer function of LIWC-22 to generate sentence fragments containing words related to each LIWC domain included in the analysis. We then analyzed changes in the clinical note before and after CD training, using the complete visit note as the unit of analysis. To calculate changes in the content characteristics of clinical notes before and after CD training, we calculated frequency scores of each LIWC category for every clinical note since this study focuses on examining the presence of words from the LIWC dictionary.

Instead of using LIWC scores based on percentages, we used frequency scores. This decision was made based on previous research indicating that CD enhances the length of clinical notes in terms of word and character count [31]. Additionally, we used frequencies to assess the presence of PCC-related language in clinical notes. Our primary interest was to capture whether clinicians used PCC-related words in their documentation, even if these words did not constitute a large proportion of the total text. By focusing on word frequencies, we aimed to mitigate the potential impact of note length inflation due to CD practices, such as copy-and-pasting or using templates [32,33]. The LIWC frequency scores are calculated by the following steps. First, we calculated LIWC scores, which are determined by the percentage of words in a text that belong to specific linguistic categories. Then, to find the frequency of each category within a clinical note, the respective LIWC percentage is multiplied by the total word count of the note. For example, an LIWC value of 1.02 for achievement indicates that the note contains 1.02 words related to achievement per the LIWC dictionary.

We used a fixed effects model that included the provider as a categorical variable. The changes in the notes before and after CD training were calculated while accounting for nesting within the therapist using individual fixed-effects models. This approach allowed us to examine whether the changes in PCC language use before and after the CD training varied across the 10 providers in our sample. To further investigate these differences, we calculated the intraclass correlation coefficient (ICC) and conducted paired sample *t* tests for each provider (Multimedia Appendix 2). The LIWC version 22 was used for dictionary-based sentiment analysis and STATA (version 17.0; Statacorp) was used for statistical analyses.

## Results

### Contextualizing LIWC Categories

The sentences generated by the Contextualizer function illustrated how the LIWC categories mapped onto the PCCP principles (see Table 2). The drives subcategories (achievement, affiliation, and power) reflected a strength-based approach by describing the positive changes made by the client; greater self-determination by feeling more in control; and interests by capturing how a client feels connected to community, people, and pets. The lifestyle subcategories (home, leisure, money, religion, and work) captured the unique details of the person’s life that are needed to individualize treatment, including their beliefs, values, and preferences which inform their personal life goals. Examples included going on a cruise as part of self-care and seeking employment. Lifestyle categories also illustrated people’s interests such as playing sports and describing their life in the community such as attending church. Physical categories (physical and wellness) demonstrated a more holistic approach to the client by paying attention to how physical health affects mental health and also to a focus on recovery by including activities that promote wellness such as yoga and health coping skills. The social referents category (family and friend) demonstrated the role family and friends play as natural supports such as going on vacation with your sister or having fun with your friends.

### Changes in LIWC Categories

Overall, there was a significant positive change in 4 of the selected LIWC categories indicating person-centeredness after the providers had been trained in CD. As shown in Table 3, the fixed effects regression analysis found the following among the 12 selected characteristics: an increased use in 4 categories, decreased use in 4 categories, and no change in use in 4 categories, while controlling for length of sessions at the therapist level. Within the drives category, we observed a significant increase in words associated with achievement (β=.774, *P*<.001, ICC=0.146) and power (β=.831, *P*<.001, ICC=0.072), with the ICC values indicating that 14.6% and 7.2% of the variance in these word categories, respectively, could be attributed to differences between therapists. In the lifestyle category, there was an increase in the use of words related to home (β=.047, *P*=.35, ICC=0.060) and work (β=.047, *P*=.12, ICC=0.285), but these changes are not statistically significant. The ICC values suggest that 6.0 and 28.5% of the variance in these word categories, respectively, could be attributed to differences between therapists. On the other hand, leisure and religion-associated words showed a significant decrease (β=−.166, *P*=.002, ICC=0.033; β=−.105, *P*<.001, ICC=0.031, respectively), while words associated with money displayed substantial increases (β=.204, *P*<.001, ICC=0.035). The ICC values for these categories indicate that 3.3%, 3.1%, and 3.5% of the variance, respectively, could be attributed to differences between therapists. In the health category, there was a notable increase in the use of physical health–related words (β=.427, *P*=.03, ICC=0.159), with 15.9% of the variance attributable to differences between therapists. In contrast, wellness-related words decreased significantly (β=−.427, *P*<.001, ICC=0.211), with 21.1% of the variance attributable to differences between therapists. In the social referents category, the use of family-related words did not show any significant change (β=−.016, *P*=.89, ICC=0.085), with 8.5% of the variance attributable to differences between therapists. However, the frequency of friend-related words decreased significantly (β=−.084, *P*=.005, ICC=0.028), with only 2.8% of the variance attributable to differences between therapists.

**Table 3.** Person-centeredness before and after collaborative documentation (CD). Coefficients were reported. Standard errors are in parentheses. β denotes coefficients of fixed-effects models. Fixed-effects estimates were based on models from the STATA module “xtreg” commands, clustered by therapist and with controls for length of session (minutes).

Category		Sample words	Frequency mean		Fixed effects		
			Before CD	After CD	ICC^a^	β (SE)	*P* value
**Drives**							
	Achievement	work, better, best, working	4.04	5.09	0.146	.774 (0.129)	<.001
	Affiliation	we, our, us, help	4.28	4.24	0.212	.086 (0.138)	.53
	Power	wn, order, allow, power	1.75	2.66	0.072	.831 (0.094)	<.001
**Lifestyle**							
	Home	home, house, room, bed	0.66	0.77	0.060	.047 (0.051)	.35
	Leisure	game, fun, play, party	0.78	0.62	0.033	−.166 (0.053)	.002
	Money	business, pay, price, market	0.20	0.41	0.035	.204 (0.035)	<.001
	Religion	god, hell, christmas, church	0.21	0.10	0.031	−.105 (0.029)	<.001
	Work	work, school, working, class	6.20	6.68	0.285	.234 (0.152)	.12
**Health**							
	Physical	medic, food, patients, eye	5.43	5.90	0.159	.427 (0.193)	.03
	Wellness	healthy, gym, supported, diet	0.93	0.59	0.211	−.416 (0.057)	<.001
**Social referents**							
	Family	parent, mother, father, baby	2.24	2.09	0.085	−.016 (0.116)	.89
	Friend	friend, boyfriend, girlfriend, dude	0.26	0.18	0.028	−.084 (0.03)	.005

a ICC: intraclass correlation coefficient.

## Discussion

### Principal Findings

The contextualizing analysis provided insight into the documentation content reflecting the selected LIWC categories and demonstrated how person-centered principles can be integrated into clinical documentation. Using LIWC categories illustrated how providers described their clients in ways that gave a sense of their lives beyond their mental health. These details included what they care about and how that related to their personal goals (getting a job, financial situation, going on a cruise, or attending church), their life beyond the clinic in the community (their home life, family and friends, and their community). The sentences also showed when clinicians used a strengths-based approach, the nature and content of their clinic notes changed in ways that moved beyond symptoms [34].

The quantitative results indicating whether there was an increase in person-centeredness of clinical documentation as indicated by relevant LIWC categories were mixed, with a significant increase in half the subcategories. The most pronounced positive increase was within the drives category, with words associated with power and achievement increasing. In terms of PCC, this indicates providers made more reference to self-determination, including how the client has made progress, and their strengths. Lifestyle categories, which include words related to hobbies and other social activities were more mixed, showing that providers were not consistent in increasing their focus on personal life goals or taking a holistic view of the client. In health, there was a significant increase in references to physical health but a decrease in references to wellness. This may reflect the increasing efforts to integrate health into their clinical interventions [35], but does not indicate a more recovery-oriented focus. Finally, in terms of social referents, there was no change in family references, which may be due to the fact that family inclusion is a common best practice of PCC [36,37], and a decrease in references to friends, often considered a source of natural supports within PCC.

Existing work has suggested that CD can improve important indicators of PCC, including service engagement and the quality of the working alliance [1,8], but research has yet not illuminated how this practice impacts care processes, including how person-centered principles are integrated into clinical interventions. Through analysis of session notes, this study found an increase in strengths-based approaches to clinical documentation following CD training, which may also reflect a shift towards interventions that emphasize self-determination. In addition to expanding the limited evidence base around the impact of CD on clinical quality, this study uniquely describes the mechanisms through which CD supports alliance building and engagement in care.

To meet the much-documented challenges of measuring PCC [38,39], a core component of health care reform, this study sought to harness the richness of clinical narrative data using a machine learning algorithm. While some studies have used NLP to study psychotherapy sessions [25], this is one of the first to mine narrative psychosocial documentation in behavioral health settings. Overall, this methodological approach has considerable potential to sample large quantities of documentation to measure different aspects of care quality, including PCC. NLP can be used both by researchers to examine how quality of care predicts clinical outcomes and by clinics to promote and document quality improvement.

Despite this potential, there are considerable challenges in calibrating algorithms so that they can accurately capture more nuanced aspects of care, such as person-centeredness. This study chose to use a well-validated dictionary designed to capture psychological concepts within narrative data. Although the study team was able to map existing LIWC categories onto established principles of PCC, the algorithm was not explicitly designed to measure these constructs, which may have contributed to the lack of positive findings within certain subcategories. Furthermore, the LIWC dictionary’s focus on single words limits the algorithm’s ability to capture more nuanced meanings that occur when words are evaluated within the larger context of surrounding phrases or sentences. This suggests the need to develop an algorithm focused specifically on PCC.

This study showed the potential for using NLP techniques to measure the quality of care within behavioral health settings. As more care standards demand that clinics demonstrate PCC [40] within mental health, there is a pressing need for feasible methods to capture this quality dimension. Being able to use at the aggregate level note data that reflects more nuanced and individualized aspects of care would help clinics document and report PCC.

In addition, our study highlights the value of clinical notes for research in behavioral health settings. Clinical notes have been harnessed for research purposes but have mainly been confined to hospital settings due to the scarcity of publicly available data [41]. The nature of psychosocial documentation differs from other clinical notes, which require different analytics and models and there is a need for publicly available data sets for analyzing psychotherapy notes in the United States. Furthermore, mental health notes often contain identifiable and sensitive information different from other clinical notes for physical illnesses, so an in-depth discussion on ethics, privacy, and deidentification and the development of techniques such as word embedding models to improve the privacy of clinical notes [42,43] for mental health notes is required.

### Limitations

The study was limited to the scope of the LIWC categories rather than an algorithm developed specifically to capture PCC and therefore, was not able to measure the concept in its entirety. Furthermore, the analysis limited itself to categories with a positive valence rather than also measuring the inverse of PCC. Although well validated, the LIWC can still fail to capture the meaning of words and mis-categorize them but as it is used more, the algorithm will continue to be trained and improved. Overall, while documentation is an important indicator of PCC, it does not directly capture the interpersonal interactions between the provider and the clinician which shape a client’s experience of PCC.

While our fixed effects model controlled for Provider ID and Date of Service, we were unable to account for potential differences in therapy modality or length of stay. In our study setting, the therapy modality primarily consisted of individual psychotherapy sessions excluding 2 notes (1 group therapy and 1 family therapy) resulting in insufficient variation to control for this variable. Additionally, our current data set did not include sufficient information on length of stay to include this variable in the model. Future research could benefit from examining the influence of therapy modality and longitudinal factors on PCC language use in clinical documentation.

It is important to acknowledge that while our study demonstrates an increase in person-centered content within clinic visit notes following the implementation of the CD, we did not directly assess whether the increased PCC in the clinic visit notes was associated with improved PCC practices. Future research should investigate the relationship between the presence of PCC in clinical notes and its impact on PCC practices and outcomes.

### Conclusion

This study is an important first step in using NLP to measure the quality of care through narrative clinical notes in behavioral health settings. We were able to identify key PCC principles within the notes using a dictionary-based approach and examine whether CD changes the way providers document with respect to PCC. This demonstrates the potential for NLP to be used by both researchers and clinics as a quality improvement tool and the importance of further developing algorithms that can capture the nuances of PCC.

## Supplementary material

10.2196/52678 Multimedia Appendix 1 Person-centeredness before and after collaborative documentation: control for negation words.

10.2196/52678 Multimedia Appendix 2 Paired sample *t* test.

## Abbreviations

CD: collaborative documentation
EHR: electronic health record
ICC: intraclass correlation coefficient
LIWC: Linguistic Inquiry and Word Count
NLP: natural language processing
PCC: person-centered care
PCCP: person-centered care planning

## References

[1] Stanhope V, Ingoglia C, Schmelter B, Marcus SC (2013). Impact of person-centered planning and collaborative documentation on treatment adherence. Psychiatr Serv.

[2] Matthews EB (2017). Integrating the electronic health record into behavioral health encounters: strategies, barriers, and implications for practice. Adm Policy Ment Health.

[3] DiCarlo R, Garcia YE, Tettegah SY, Garcia YE (2016). Emotions, Technology, and Health.

[4] Institute of Medicine (US) Committee on Quality of Health Care in America (2001). Crossing the Quality Chasm: A New Health System for the 21st Century.

[5] Tondora J, Miller R, Slade M, Davidson L (2014). Partnering for Recovery in Mental Health: A Practical Guide to Person-Centered Planning.

[6] Tondora J, Pocklington S, Osher D, Davidson L (2005). Implementation of person-centered care and planning: from policy to practice to evaluation.

[7] Schwarz J, Bärkås A, Blease C (2021). Sharing clinical notes and electronic health records with people affected by mental health conditions: scoping review. JMIR Ment Health.

[8] Matthews EB (2020). Computer use in mental health treatment: understanding collaborative documentation and its effect on the therapeutic alliance. Psychotherapy (Chic).

[9] O’Neill S, Blease C, Delbanco T (2021). Open notes become law: a challenge for mental health practice. Psychiatr Serv Am Psychiatric Assoc.

[10] Agency for Planning and Evaluation Certified Community Behavioral Health Clinics Demonstration Program: Report to Congress, 2019.

[11] Hyun S, Johnson SB, Bakken S (2009). Exploring the ability of natural language processing to extract data from nursing narratives. Comput Inform Nurs.

[12] Haselden M, Dixon LB, Overley A (2018). Giving back to families: evidence and predictors of persons with serious mental illness contributing help and support to families. Community Ment Health J.

[13] Butler JM, Gibson B, Patterson OV (2022). Clinician documentation of patient centered care in the electronic health record. BMC Med Inform Decis Mak.

[14] Abbe A, Grouin C, Zweigenbaum P, Falissard B (2016). Text mining applications in psychiatry: a systematic literature review. Int J Methods Psychiatr Res.

[15] Edgcomb JB, Zima B (2019). Machine learning, natural language processing, and the electronic health record: innovations in mental health services research. Psychiatr Serv.

[16] Dreisbach C, Koleck TA, Bourne PE, Bakken S (2019). A systematic review of natural language processing and text mining of symptoms from electronic patient-authored text data. Int J Med Inform.

[17] Mascio A, Kraljevic Z, Bean D (2020). Comparative analysis of text classification approaches in electronic health records.

[18] Afshar M, Phillips A, Karnik N (2019). Natural language processing and machine learning to identify alcohol misuse from the electronic health record in trauma patients: development and internal validation. J Am Med Inform Assoc.

[19] Carson NJ, Mullin B, Sanchez MJ (2019). Identification of suicidal behavior among psychiatrically hospitalized adolescents using natural language processing and machine learning of electronic health records. PLoS ONE.

[20] Hammond KW, Ben‐Ari AY, Laundry RJ, Boyko EJ, Samore MH (2015). The feasibility of using large‐scale text mining to detect adverse childhood experiences in a VA‐treated population. J Trauma Stress.

[21] Rajendran S, Topaloglu U (2020). Extracting smoking status from electronic health records using NLP and deep learning. AMIA Jt Summits Transl Sci Proc.

[22] McCoy TH, Castro VM, Cagan A, Roberson AM, Kohane IS, Perlis RH (2015). Sentiment measured in hospital discharge notes is associated with readmission and mortality risk: an electronic health record study. PLoS ONE.

[23] Zerden L de S, Lombardi BM, Richman EL, Fraher EP, Shoenbill KA (2021). Harnessing the electronic health record to advance integrated care. Fam Syst Health.

[24] Rahimian M, Warner JL, Jain SK, Davis RB, Zerillo JA, Joyce RM (2019). Significant and distinctiven-grams in oncology notes: a text-mining method to analyze the effect of opennotes on clinical documentation. JCO Clin Cancer Inform.

[25] Atkins DC, Steyvers M, Imel ZE, Smyth P (2014). Scaling up the evaluation of psychotherapy: evaluating motivational interviewing fidelity via statistical text classification. Impl Sci.

[26] Blease C, Torous J, Hägglund M (2020). Does patient access to clinical notes change documentation?. Front Public Health.

[27] Alpert JM, Morris BB, Thomson MD, Matin K, Sabo RT, Brown RF (2019). Patient access to clinical notes in oncology: a mixed method analysis of oncologists’ attitudes and linguistic characteristics towards notes. Patient Educ Couns.

[28] (2023). MTM Services.

[29] Boyd RL, Ashokkumar A, Seraj S, Pennebaker JW (2022). The development and psychometric properties of LIWC-22.

[30] Klein D (2018). Implementing a general framework for assessing interrater agreement in stata. Stata J.

[31] Yoo N, Matthews E, Baslock D, Stanhope V (2023). Impact of collaborative documentation on completeness and length of clinical notes in behavioral health settings. Psychiatr Serv.

[32] Thornton JD, Schold JD, Venkateshaiah L, Lander B (2013). Prevalence of copied information by attendings and residents in critical care progress notes. Crit Care Med.

[33] Wang MD, Khanna R, Najafi N (2017). Characterizing the source of text in electronic health record progress notes. JAMA Intern Med.

[34] Braun MJ, Dunn W, Tomchek SD (2017). A pilot study on professional documentation: do we write from a strengths perspective?. Am J Speech Lang Pathol.

[35] Scharf DM, Eberhart NK, Schmidt N (2013). Integrating primary care into community behavioral health settings: programs and early implementation experiences. Psychiatr Serv.

[36] Frampton SB, Giuliano M (2023). Patient-centered care: the North Star to guide us during uncertainty into a better day. Int J Qual Health Care.

[37] Boise L, White D (2004). The family’s role in person-centered care: practice considerations. J Psychosoc Nurs Ment Health Serv.

[38] Stanhope V, Baslock D, Tondora J, Jessell L, Ross AM, Marcus SC (2021). Developing a tool to measure person-centered care in service planning. Front Psychiatry.

[39] Burgers JS, van der Weijden T, Bischoff E (2021). Challenges of research on person-centered care in general practice: a scoping review. Front Med.

[40] (2022). Person-centered service planning guidelines for Medicaid managed care organizations, local departments of social services, and health homes. New York State Department of Health.

[41] Zhou N, Wu Q, Wu Z, Marino S, Dinov ID (2022). DataSifterText: partially synthetic text generation for sensitive clinical notes. J Med Syst.

[42] Abdalla M, Abdalla M, Rudzicz F, Hirst G (2020). Using word embeddings to improve the privacy of clinical notes. J Am Med Inform Assoc.

[43] Abdalla M, Abdalla M, Hirst G, Rudzicz F (2020). Exploring the privacy-preserving properties of word embeddings: algorithmic validation study. J Med Internet Res.

