# Supplementary material for: The Impact of Collaborative Documentation on Person-Centered Care: Textual Analysis of Clinical Notes
Source: JMIR Med Inform. 2024 Sep 20;12:e52678. doi: 10.2196/52678 (PMC11429664; doi:10.2196/52678)
Supplement: Multimedia Appendix 1 [file medinform-v12-e52678-s001.docx]

Table S1. Person-centeredness before and after CD: Control for Negation Words

| Category | Sample words | ICC | β (SE) | p-value |
| --- | --- | --- | --- | --- |
| Drives |  |  |  |  |
| Achievement | work, better, best, working | 0.153 | 0.754 (0.129) | <.001 |
| Affiliation | we, our, us, help | 0.218 | 0.003 (0.136) | 0.981 |
| Power | own, order, allow, power | 0.075 | 0.795 (0.094) | <.001 |
| Lifestyle |  |  |  |  |
| Home | home, house, room, bed | 0.057 | 0.038 (0.051) | 0.45 |
| Leisure | game, fun, play, party | 0.038 | -0.182 (0.053) | 0.001 |
| Money | business, pay, price, market | 0.037 | 0.198 (0.036) | <.001 |
| Religion | god, hell, Christmas, church | 0.031 | -0.108 (0.029) | <.001 |
| Work | work, school, working, class | 0.281 | 0.177 (0.152) | 0.244 |
| Health |  |  |  |  |
| Physical | medic, food, patients, eye, | 0.192 | 0.3 (0.191) | 0.116 |
| Wellness | healthy, gym, supported, diet | 0.215 | -0.423 (0.057) | <.001 |
| Social referents |  |  |  |  |
| Family | parent, mother, father, baby | 0.072 | -0.064 (0.115) | 0.579 |
| Friend | friend, boyfriend, girlfriend, dude | 0.030 | -0.089 (0.03) | 0.003 |

Note: Coefficients were reported. Standard errors are in parentheses. β denotes coefficients of fixed-effects models. Fixed-effects estimates were based on models from the Stata module “xtreg” commands, clustered by therapist and with controls for length of session (minutes) and negation word frequency
