# Supplementary material for: The Impact of Collaborative Documentation on Person-Centered Care: Textual Analysis of Clinical Notes
Source: JMIR Med Inform. 2024 Sep 20;12:e52678. doi: 10.2196/52678 (PMC11429664; doi:10.2196/52678)
Supplement: Multimedia Appendix 2 [file medinform-v12-e52678-s002.docx]

Table S2. Paired Sample T-Test

|  | Sample 1 | | | Sample 2 | | | Sample 3 | | | Sample 4 | | | Sample 5 | | |
| --- | --- | --- | --- | --- | --- | --- | --- | --- | --- | --- | --- | --- | --- | --- | --- |
|  | **Pre-CD** | **Post-CD** | ***t* (*p*)** | **Pre-CD** | **Post-CD** | ***t* (*p*)** | **Pre-CD** | **Post-CD** | ***t* (*p*)** | **Pre-CD** | **Post-CD** | ***t* (*p*)** | **Pre-CD** | **Post-CD** | ***t* (*p*)** |
| Category |  |  |  |  |  |  |  |  |  |  |  |  |  |  |  |
| Drives |  |  |  |  |  |  |  |  |  |  |  |  |  |  |  |
| Achievement | 2.75 | 4.08 | -1.43 (0.18) | 4.38 | 6.10 | -2.55 (0.02) | 4.67 | 6.93 | -2.77 (0.02) | 2.08 | 3.08 | -1.3 (0.22) | 3.90 | 4.70 | -0.7 (0.5) |
| Affiliation | 4.17 | 4.25 | -0.08 (0.94) | 4.81 | 4.05 | 1.27 (0.22) | 5.20 | 6.46 | -2.31 (0.04) | 3.69 | 3.46 | 0.24 (0.82) | 2.30 | 3.70 | -1.5 (0.17) |
| Power | 2.50 | 3.41 | -1.25 (0.24) | 1.81 | 3.43 | -3.48 (0) | 0.93 | 1.93 | -1.9 (0.08) | 2.23 | 1.31 | 1.8 (0.1) | 1.30 | 3.70 | -1.59 (0.15) |
| Lifestyle |  |  |  |  |  |  |  |  |  |  |  |  |  |  |  |
| Home | 0.08 | 0.83 | -3.45 (0.01) | 0.28 | 0.86 | -2.04 (0.05) | 0.87 | 0.60 | 0.81 (0.43) | 0.46 | 1.00 | -1.34 (0.21) | 0.10 | 1.70 | -2.51 (0.03) |
| Leisure | 0.25 | 0.08 | 0.99 (0.34) | 0.43 | 0.48 | -0.24 (0.81) | 1.86 | 0.66 | 4.05 (0) | 0.08 | 0.77 | -2.63 (0.02) | 1.20 | 0.70 | 0.59 (0.57) |
| Money | 0.42 | 0.00 | 2.16 (0.05) | 0.48 | 0.33 | 0.46 (0.65) | 0.00 | 0.33 | -2.65 (0.02) | 0.31 | 0.23 | 0.43 (0.67) | 0.10 | 0.00 | 1 (0.34) |
| Religion | 0.92 | 0.17 | 1.43 (0.18) | 0.14 | 0.05 | 0.81 (0.43) | 0.27 | 0.20 | 0.37 (0.72) | 0.00 | 0.08 | -1 (0.34) | 0.00 | 0.00 | () |
| Work | 5.58 | 5.92 | -0.31 (0.76) | 3.24 | 4.90 | -1.89 (0.07) | 5.40 | 6.00 | -0.83 (0.42) | 9.85 | 11.77 | -2.12 (0.06) | 5.00 | 8.70 | -2.11 (0.06) |
| Health |  |  |  |  |  |  |  |  |  |  |  |  |  |  |  |
| Physical | 4.00 | 4.08 | -0.05 (0.96) | 7.81 | 7.76 | 0.03 (0.98) | 7.53 | 7.27 | 0.21 (0.83) | 3.38 | 3.23 | 0.22 (0.83) | 3.50 | 2.20 | 1.16 (0.28) |
| Wellness | 0.58 | 0.50 | 0.22 (0.83) | 0.86 | 0.43 | 1.63 (0.12) | 2.13 | 1.40 | 1.44 (0.17) | 0.54 | 0.54 | 0 (1) | 1.00 | 0.00 | 1.68 (0.13) |
| Social referents |  |  |  |  |  |  |  |  |  |  |  |  |  |  |  |
| Family | 1.00 | 1.25 | -0.53 (0.61) | 2.76 | 2.24 | 1.02 (0.32) | 1.20 | 1.00 | 0.43 (0.68) | 3.92 | 2.69 | 1.89 (0.08) | 0.50 | 2.40 | -3.77 (0) |
| Friend | 0.25 | 0.42 | -0.56 (0.59) | 0.43 | 0.14 | 1.1 (0.29) | 0.13 | 0.40 | -1.29 (0.22) | 0.08 | 0.00 | 1 (0.34) | 0.10 | 0.20 | -0.43 (0.68) |
|  | **Sample 6** | | | **Sample 7** | | | **Sample 8** | | | **Sample 9** | | | **Sample 10** | | |
|  | **Pre-CD** | **Post-CD** | ***t* (*p*)** | **Pre-CD** | **Post-CD** | ***t* (*p*)** | **Pre-CD** | **Post-CD** | ***t* (*p*)** | **Pre-CD** | **Post-CD** | ***t* (*p*)** | **Pre-CD** | **Post-CD** | ***t* (*p*)** |
| Category |  |  |  |  |  |  |  |  |  |  |  |  |  |  |  |
| Drives |  |  |  |  |  |  |  |  |  |  |  |  |  |  |  |
| Achievement | 3.70 | 2.55 | 2.19 (0.04) | 2.33 | 4.16 | -3.78 (0) | 4.34 | 5.67 | -1.1 (0.38) | 4.22 | 4.33 | -0.14 (0.89) | 5.72 | 6.57 | -0.59 (0.57) |
| Affiliation | 2.15 | 2.25 | -0.16 (0.88) | 8.16 | 6.76 | 1.62 (0.11) | 5.67 | 3.34 | 0.62 (0.6) | 3.72 | 1.55 | 3.35 (0) | 3.22 | 3.57 | -0.34 (0.74) |
| Power | 1.20 | 1.45 | -0.57 (0.58) | 1.13 | 1.49 | -1.4 (0.17) | 1.66 | 2.33 | -0.5 (0.67) | 2.28 | 2.78 | -0.77 (0.45) | 1.21 | 2.36 | -1.69 (0.12) |
| Lifestyle |  |  |  |  |  |  |  |  |  |  |  |  |  |  |  |
| Home | 0.30 | 0.25 | 0.33 (0.75) | 0.81 | 0.86 | -0.18 (0.86) | 1.66 | 1.33 | 1 (0.42) | 0.11 | 0.72 | -3.32 (0) | 1.50 | 0.50 | 2.25 (0.04) |
| Leisure | 0.95 | 0.20 | 2.51 (0.02) | 0.57 | 0.59 | -0.1 (0.92) | 0.00 | 1.00 | -1.73 (0.23) | 0.61 | 0.83 | -0.81 (0.43) | 0.79 | 0.43 | 1.24 (0.24) |
| Money | 0.05 | 0.45 | -1.45 (0.16) | 0.03 | 0.00 | 1 (0.32) | 0.00 | 0.00 | N/A | 0.33 | 0.11 | 1 (0.33) | 0.07 | 0.71 | -1.8 (0.1) |
| Religion | 0.00 | 0.30 | -2.35 (0.03) | 0.03 | 0.00 | 1 (0.32) | 0.00 | 0.00 | N/A | 0.22 | 0.06 | 1 (0.33) | 0.07 | 0.14 | -0.44 (0.67) |
| Work | 5.55 | 3.50 | 2.86 (0.01) | 5.49 | 6.08 | -1.03 (0.31) | 5.67 | 7.67 | -2 (0.18) | 2.83 | 3.56 | -0.84 (0.41) | 8.29 | 8.28 | 0 (1) |
| Health |  |  |  |  |  |  |  |  |  |  |  |  |  |  |  |
| Physical | 3.20 | 4.95 | -1.97 (0.06) | 1.84 | 2.76 | -1.58 (0.12) | 5.67 | 2.33 | 2 (0.18) | 6.94 | 8.11 | -1.33 (0.2) | 11.21 | 5.57 | 3.42 (0) |
| Wellness | 0.70 | 0.25 | 1.63 (0.12) | 0.11 | 0.24 | -1.04 (0.3) | 0.67 | 0.34 | 1 (0.42) | 0.72 | 0.83 | -0.29 (0.78) | 4.29 | 0.43 | 4.76 (0) |
| Social referents |  |  |  |  |  |  |  |  |  |  |  |  |  |  |  |
| Family | 1.65 | 2.70 | -1.8 (0.09) | 4.54 | 1.43 | 3.97 (0) | 3.67 | 1.66 | 0.62 (0.6) | 1.89 | 0.56 | 2.83 (0.01) | 1.50 | 0.71 | 1.18 (0.26) |
| Friend | 0.25 | 0.15 | 0.46 (0.65) | 0.30 | 0.11 | 1.65 (0.11) | 0.00 | 0.34 | -1 (0.42) | 0.11 | 0.00 | 1 (0.33) | 0.07 | 0.00 | 1 (0.34) |
